# Loss of Skeletal Muscle Mass and Intracellular Water as Undesired Outcomes of Weight Reduction in Obese Hyperglycemic Women: A Short-Term Longitudinal Study

**DOI:** 10.3390/ijerph19021001

**Published:** 2022-01-17

**Authors:** Jolanta Zalejska-Fiolka, Anna Birková, Tomasz Wielkoszyński, Beáta Hubková, Beata Szlachta, Rafał Fiolka, Urszula Błaszczyk, Aleksandra Kuzan, Andrzej Gamian, Mária Mareková, Michał Toborek

**Affiliations:** 1 Department of Biochemistry, Faculty of Medical Science in Zabrze, Medical University of Silesia, 40-055 Katowice, Poland; jzalejskafiolka@sum.edu.pl (J.Z.-F.); beata.szlachta@icloud.com (B.S.); ublaszczyk@sum.edu.pl (U.B.); 2 Department of Clinical and Medical Biochemistry, Pavol Jozef Šafárik University, 040 11 Košice, Slovakia; maria.marekova@upjs.sk; 3 Higher School of Strategic Planning, 41-303 Dąbrowa Górnicza, Poland; t.wielkoszynski@wielkoszynski.pl; 4 Doctoral School, Faculty of Medical Science in Zabrze, Medical University of Silesia, 40-055 Katowice, Poland; fiolkarafal@gmail.com; 5 Department of Medical Biochemistry, Wrocław Medical University, 50-367 Wrocław, Poland; aleksandra.kuzan@umed.wroc.pl; 6 Institute of Immunology and Experimental Therapy, 53-114 Wrocław, Poland; andrzej.gamian@hirszfeld.pl; 7 Department of Biochemistry and Molecular Biology, University of Miami School of Medicine, Miami, FL 33136, USA; mtoborek@med.miami.edu

**Keywords:** anthropometric parameters, bioimpedance, extracellular body water (ECW), hyperglycemia, intracellular body water (ICW), total body water (TBW), free fat mass (FFM), visceral fat area (VFA), weight loss

## Abstract

The current study is focused on the influence of hyperglycemia on weight loss in obese premenopausal women. Specifically, the study evaluated the impact of a six-month individualized low-calorie diet combined with moderate exercise on weight reduction and glucose metabolism in obese women with normoglycemia compared to obese women with moderate hyperglycemia. The results indicated that patients with normoglycemia achieved a successful weight loss, which was connected to a decrease in adipose tissue and reflected by diminished content of visceral fat area (VFA) and percent body fat. In contrast, weight reduction in patients with hyperglycemia was connected not only to the loss of VFA but also to undesired decrease in skeletal muscle mass as well as intracellular and total body water. These unfavorable outcomes were observed despite normalization of glucose metabolism reflected by statistically significant lowering glucose, fructosamine, advanced glycation end-products, and HOMA-IR levels. Overall, the obtained results indicate the importance of the measurement of the carbohydrate profile in obese women and the need for an early introduction of weight reduction strategies before the development of hyperglycemia.

## 1. Introduction

Background: The prevalence of overweightness and obesity is increasing rapidly, especially in developing countries, and affects approximately 650 million people worldwide [1]. In Poland, approximately 61% of men and 50% of women are overweight or obese [2]. There is also an equally high rate of increase in the incidence of Type 2 diabetes (T2D) worldwide. The World Health Organization Global Report on diabetes indicates that the number of adults living with T2D has almost quadrupled to 422 million people since 1980 [3] and is expected to increase to 693 million by 2045 [4]. The disease is characterized by high blood glucose and insulin levels due to tissue insulin resistance. There is a strong association between obesity and T2D, with more than 80% of obese individuals affected by this disease.

Health risks associated with obesity differ substantially depending on the distribution of fat tissue. In contrast to the accumulation of peripheral subcutaneous fat, excessive visceral fat (i.e., abdominal obesity) is strongly associated with obesity-related complications, including insulin resistance, hyperinsulinemia, hyperglycemia, dyslipidemia, and hypertension [5]. Waist circumference, an estimate of abdominal obesity, is positively associated with insulin resistance and the progression from impaired glucose tolerance to T2D [6,7]. Therefore, an intervention that specifically targets abdominal obesity and insulin resistance has important implications for diabetes prevention [8].

While abdominal obesity is more common in men, it is associated with more health risks in women. A larger waist circumference and waist-to-hip ratio (WHR) conferred a greater risk of cardiovascular disorders (CVDs) in women than in men. In addition, the WHR ratio was more strongly associated with the risk of CVDs than the body mass index (BMI) in women [9,10]. Several studies have also demonstrated that T2D affects women disproportionately [11]. Women with T2D have poorer glycemic control and, when treated, are less likely to reach the desired normalization of glycated hemoglobin (HbA1c) compared to men [12,13,14].

Weight loss programs that employ effective and safe tools are critical to prevent the development of T2D, cardiovascular diseases, and various other diseases, including cancer [15]. The foundation of a successful weight loss strategy should be lifestyle changes, including high-quality, appropriate, and individualized diet and increased physical activity [16,17]. Indeed, a personalized diet combined with regular check-ups is considered to be an effective strategy to improve adherence to the recommendations and avoid body weight regain, helping with permanent lifestyle changes and gaining greater long-term benefits [18]. Adding physical activity to a hypocaloric, individualized diet is important to reduce fat mass only and preserve muscle mass during weight loss [19].

Visceral fat mass is closely related to the metabolic dysregulations in obesity, and excess of visceral fat leads to the development of insulin resistance and fat accumulation in internal organs such as liver, heart, pericardium, skeletal muscles, vascular walls, or blood vessels, which then trigger to the pathophysiology of metabolic diseases. Therefore, a decrease in fat mass during body mass reduction is associated with the improvement of metabolic alterations [20,21,22,23]. On the other hand, a loss of fat-free mass, including muscle mass, could be associated with unfavorable results including development of certain pathologies such as sarcopenia [21,24].

The mechanisms participating in a decrease in muscle mass during weight reduction are not fully understood. Evidence indicates that a short-term (up to 21 days) calorie restriction (30–40% energy deficit/day) can decrease the postprandial rate of muscle protein synthesis and reduce the basal rate of muscle protein synthesis [25]. On the other hand, it was indicated that a prolonged moderate calorie restriction and 5–10% weight loss increased the rate of muscle protein synthesis [26]. Overall, the loss of muscle mass during a prolonged moderate calorie restriction is believed to be mediated by increased muscle proteolysis rather than suppressed muscle protein synthesis, and it is viewed as an undesirable effect. It appears that increased physical activity during weight loss may protect against this unfavorable process [27]. The results of the current study indicate that other factors that affect the type of tissue being reduced during weight loss should also be taken into account. One of such factors could be untreated hyperglycemia/impaired glucose tolerance.

The objective of the present study was to evaluate the impact of hyperglycemia on weight loss in premenopausal and hyperglycemic women with abdominal obesity. We hypothesized that elevated glucose levels and impaired glucose tolerance in obese women on personalized diets and exercise programs can affect weight reduction and loss of non-fat tissues. Due to gender-related metabolic differences, we focused our research on premenopausal women. While obese women with normoglycemia achieved a successful weight loss, which was connected to a decrease in adipose tissue and reflected by diminished content of visceral fat area (VFA), weight reduction in obese women with hyperglycemia was associated not only with the loss of VFA but also with an undesired decrease in skeletal muscle mass as well as intracellular and total body water. The conducted research shows that hyperglycemia/impaired glucose tolerance may be one of the factors causing the loss of free fat mass tissue during weight reduction.

## 2. Methods

### 2.1. Study Design

The study was a prospective, one-center study. Patient recruitment and weight reduction program started in October 2016 and lasted 12 months overall. All volunteers declared their willingness to participate in the 6-month weight reduction program that consisted of a personalized low-calorie dietary program combined with physical activity and a continuous health education program that was focused on maintenance of a balanced and individualized low-calorie diet. Every month, patients took part in follow-up visits.

The study was approved by the Ethics Committee of the Medical University of Silesia in Katowice, Poland (No. KNW/0022/KB1/19/I/16). In accordance with the provisions of the abovementioned application to the ethics committee, each participant of the study gave informed consent to participate in the experiment and to publish the results of the research.

### 2.2. Participants

**Figure 1:**
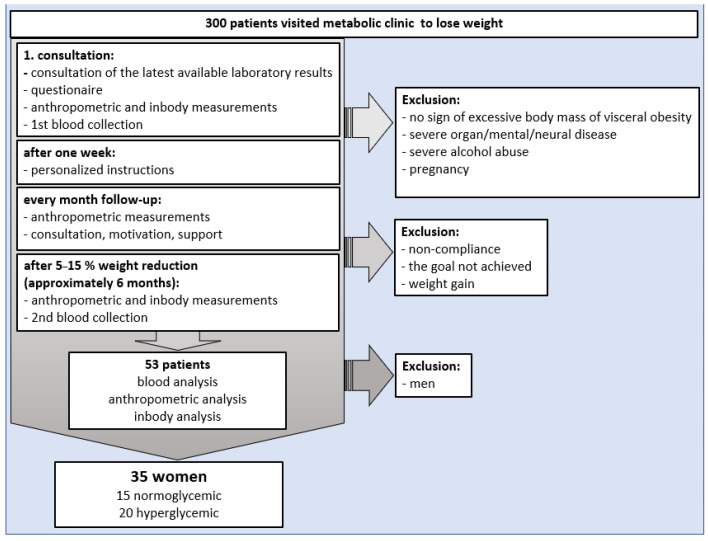
The flowchart of the study design.

Thirty-five obese women out of a total of 300 patients from the Metabolic Clinic in Miasteczko Śląskie, Poland qualified for the study (Figure 1). All patients entered the weight reduction program based on the reports indicating improving lipid and glucose levels after weight loss [28]. All women were diagnosed as obese on the basis of medical examination (body mass index (BMI), body fat mass (BFM), and visceral fat tissue (VFA). All women were characterized by BMI indicating obesity.

On the first visit, all patients were evaluated for biochemical markers, including the carbohydrate and lipid profiles. Out of 35 participants involved in the program, 15 women were normoglycemic and 20 were hyperglycemic, and none of them were taking any medications known to affect glucose and lipid metabolism. Inclusion criteria for study participants were body mass index (BMI) >30 kg/m^2^, no pharmacological treatment affecting lipid and glucose metabolism, and informed consent to participate in the study. In addition, study exclusion criteria were lack of consent to participate in the study, severe hepatic, renal, respiratory, or circulatory insufficiency, alterations of consciousness, treatment-resistant depression, chronic alcohol abuse, pregnancy, history of a severe nervous system injury, implanted cardiac pacemaker, and pharmacological treatment affecting glucose and lipid metabolism.

### 2.3. Classification of Patients into Study Groups

Study participants were categorized into two groups based on their plasma glucose levels when entering the study:

Group NG: The normoglycemic group, normoglycemic patients (15 females) with a fasting glucose level <100 mg/dL.

Group HG: The hyperglycemic group, hyperglycemic patients (20 females) with a fasting glucose level above reference values >100 mg/dL.

### 2.4. The First and Follow-Up Visit Plan, Diet Composition and Physical Activity Intervention, Variables, and Procedures to Avoid Study Bias

The patients were advised to attend one personalized consultation per month for six months. On the first visit, the following data were collected: (a) history of obesity, (b) food preferences and food habits, (c) number and quality of meals consumed per day, (d) number and quality of liquids consumed per day, (e) health problems, (f) biochemical parameters from a recent blood test, and (g) physical activity. The questionnaire revealed that patients ate unhealthily and irregularly as well as led sedentary lifestyles before starting the weight loss program.

During the first visit performed by a doctor and a dietitian (1 h/patient), the following parameters were measured based on performance protocols: (a) height; (b) body weight and body composition (InBody S10, Biospace, Cerritos, CA, United States); and (c) the individual basic metabolic rate (BMR). Based on this information, the total daily caloric demand (DCD) and daily energy deficit (calculated as 15% of the total energy of DCD) were determined for each patient.

One week after the first consultation, all patients received personalized instructions in an easily readable table about nutrition and diet composition according to recommendations by the Polish National Food and Nutrition Institute (Polish acronym, IŻŻ) and the World Health Organization, including (a) a seven-day menu adapted to taste preferences of each individual, (b) recommendations of food and liquid quantities and the numbers of meals per day (five times a day and no unhealthy snacks), (c) list of recommended and non-advised food products, and (d) the list of vegetables and fruits with glycemic index (IG) with recommendation that one portion could be eaten as a snack per day if necessary. The participants were advised to implement physical activity for a minimum of 30–40 min three times per week. The personalized diet was established for each patient and contained all the necessary ingredients, as shown in Table 1.

Every month, during the follow-up visits also performed by a doctor and a dietitian, patients were weighed, and their waist and hip circumferences were measured. Patients were motivated to continue the program and received support in the form of training in calculating calorie intake and the quality of food products. Weight reduction was monitored until achieving a healthy body weight or reduction by 5–15% over initial weight and lasted six months on average.

During the first and the last visit, fasting blood was collected from the vein of the elbow flexion in quantity of 7 mL (5 mL to obtain serum and 2 mL to obtain plasma), according to approval by the Ethics Committee application. Obtained data were collected in the Department of Biochemistry Faculty of Medical Science in Zabrze Medical University of Silesia, Katowice, Poland. To avoid bias, all the obtained data was anonymized. For biochemical tests and statistical analysis, the samples were numbered (Figure 1).

### 2.5. Biochemical Analysis

All procedures followed the good laboratory practice (GLP). Fasting blood samples were collected to the serum clot (5 mL) and EDTA (2 mL) tubes (Vacutainer, Becton Dickinson) during the first and the last visit. After clotting (ca. 30 min), blood was centrifuged (10 min, 3000 rpm), and the aliquoted serum samples were frozen in −80 degrees Celsius. The biochemical analyses were carried out in blinded samples by experienced scientific and technical staff of the Department of Biochemistry. Individual biochemical markers (except HbA1c) were measured in one repetition and one analytical series after sample collection was completed for all patients. All analytical methods were under continuous intralaboratory quality control and met the criteria of the external (interlaboratory) controls organized by the Central Center for Quality Testing in Laboratory Diagnostics in Łódź (Łódź, Poland) and the Labquality (Helsinki, Finland).

Serum glucose, fructosamine, and glycated hemoglobin (HbA1c) were determined using a biochemical analyzer (Miura 200 DA) (I.S.E. S.r.l., Guidonia Montecelio, Italy). Glucose concentration was assayed by the glucose oxidase method. The fructosamine assay was based on nitro blue tetrazolium reduction method (both reagents from Bisystems, Barcelona, Spain). HbA1c was determined in EDTA whole blood samples by the latex-enhanced immunoturbidimetry method (Human GmbH, Wiesbaden, Germany) and serum insulin by the INS-IRMA kits (KIP1251-KIP1254, DIA Source Immuno Assays S.A., Louvain, Belgium). Repeatability (within-run precision coefficients of variation) for glucose, fructosamine, HbA1c, and insulin were 0.6%, 2.1%, 1.4%, and 4.1%, respectively. Reproducibility (between-run precision coefficients of variation) for the abovementioned parameters were 1.6%, 3.9%, 3.1%, and 7.7%, respectively. The homeostatic model assessment for insulin resistance (HOMA-IR) was calculated as (fasting insulin concentration (µIU/m) × fasting glucose concentration (mg/dL))/405.

Concentrations of the advanced glycation end-products (AGE) in serum were determined in the Department of Medical Biochemistry, Wrocław Medical University, Poland by the competitive ELISA method according to Indyk et al. [29]. Briefly, 96-well MaxiSorp plates (Nunc) were coated with synthetic high molecular mass AGE adduct (HMW-AGE) based on myoglobin, obtained by high-temperature microwave synthesis, and incubated for 4 h at 37 °C. The plates were then blocked with 10% skim milk for 18 h at 4 °C. Diluted serum samples were subsequently incubated overnight at 55 °C with proteinase K from Tritirachium album (0.3 mg/mL, Sigma Aldrich, St. Louis, MI, USA) and heated for 20 min at 116 °C to denature the proteinase, cooled, centrifuged for 15 min at 15,000× *g*, and the supernatants were diluted twice with phosphate-buffered saline (PBS). Next, the samples were incubated with noncommercial monoclonal antibodies IgE anti-MAGE, and 100 μL of the mixtures were distributed to wells of the HMW-AGE-coated plate, followed by incubation at room temperature for 1.5 h. In parallel, synthetic low molecular mass AGE (LMW-AGE) solutions were incubated with antibody and distributed to wells for the preparation of a standard curve. The LMW-AGE was obtained by high-temperature microwave synthesis and isolated by gel filtration on the HW-40S and P2 bed. After incubation, plates were washed three times in PBS with 0.05% Tween 20 (PBST). Next, the reaction was carried out with secondary, horseradish peroxidase (HRP)-conjugated, anti-mouse IgE polyclonal antibody diluted 1:7000 (AP21482HR-N, OriGene Technologies, Inc., Rockville, MD, USA) for 2.5 h at room temperature, followed by washing the plates again three times with PBST. The HRP enzymatic activity bound to solid phase was assayed by a substrate solution containing o-phenylenediamine and hydrogen peroxide (Sigma Aldrich, St. Louis, MI, USA) in citrate buffer (100 μL/well). Absorbance was measured at 450 nm in the EnSpire Manager microplate reader. Concentrations of the AGE-10 were calculated from the standard curve prepared. Repeatability and reproducibility of this method were 4.5% and 7.9%, respectively. The methodology has been patented by Institute of Immunology and Experimental Therapy of the Polish Academy of Science [30].

### 2.6. Body Mass Composition Using Bioimpedance Analysis

Bioimpedance was selected for body mass composition measurement as a fast and efficient method with high accuracy [31]. Body mass composition parameters, such as arm circumference (AC), arm muscle circumference (AMC), visceral fat area (VFA), percent body fat (PBF), free fat mass (FFM), skeletal muscle mass (SMM), protein (an indicator of body nutrition), bone mineral content (BMC), total body water (TBW), intracellular body water (ICW), and extracellular body water (ECW) were analyzed during the first and the last visit (twice for each patient) using a professional body composition analyzer by bioelectrical impedance spectroscopy (InBody S10, Biospace, Cerritos, CA, USA). Resistance (R) of arms, trunk, and legs was measured after 3 h of fasting. To avoid measurement errors, the results are automatically calculated on the basis of 30 impedance measurements using six different frequencies (1 kHz, 5 kHz, 50 kHz, 250 kHz, 500 kHz, 1000 kHz) of each of the five parts of the body (right and left upper limb, torso, right and left lower limb) with an eight-polar tactile-electrode impedance meter, two of each which were in contact with the middle finger and the thumb on each arm and around the ankle of each foot. Each time, the same person performed the analysis for a given patient. Because it was a short-term study (6 months) conducted on adult women, aging and growth were not considered factors affecting the results. The device does not require calibration before measurement. The device certificates guarantee reliability, reproducibility, and calibration: the InBodyS10 analyzer had the following certificates: International ISO 9001: 2015; International ISO 13485: 2016; Medical certificate EN60601-1; Medical certificate EN60601-1-2 and CE MDD (Directive 93/42/EEC). Because body position, hydration status, consumption of food and beverages, ambient air and skin temperature, and recent physical activity affect bioimpedance results, we created the measurement protocol for standardization and calibration. The bioimpedance measurement protocol included (a) age, sex, height, body mass; (b) longitudinal follow-up was performed at the same slot, and the menstrual cycle was considered (the study was not conducted during menstruation period; (c) fasting 3 h was required; (d) no alcohol intake for 12 h; (e) no medications that affect the water balance measurement (hormones, diuretics, steroids); (f) no metallic accessories or magnetic targets; (g) the patient had to remove heavy clothes and jewelry; (h) the study was not conducted after abruptly standing up; (i) the room temperature was 20–25 degrees C during measurement. After half an hour, the analysis was performed for each patient who came to the clinic, and all the subjects were always in the upright position and had to be standing for at least 5 min before taking the measurement. The repeatability of the measurements expressed as percentage coefficients of variation was determined on the basis of 12 measurements performed on the same person in one day. All the assessed parameters were characterized by the coefficients of variation below 3%.

### 2.7. Statistical Analysis

The results are presented before and after the 6-month weight reduction program. The differences between these values are calculated as delta (Δ) and/or percentage change (Δ%). All results are shown as mean ± SD. Statistical analysis was performed with SPSS Statistics 22 (IBM, Armonk, NY, USA). The Kolmogorov–Smirnov test was used for normality testing. A two-sample *t*-test was used to determine differences of clinical parameters between groups and between values within groups before and after dietary intervention, assuming or not assuming equal variances based on Levene’s test for equality of variances. For parameters with non-normal distribution, the Mann–Whitney test was used to determine differences between groups, and the Wilcoxon signed-rank test was used to determine differences within groups before and after dietary intervention. The Pearson correlation coefficient expressed the strength and direction of the linear relationship between the two parameters.

Statistical significance was assumed at *p*-value of <0.05.

## 3. Results

### 3.1. Characteristics of Participants and Study Limitation

Despite the admission of 300 eligible patients to the program, compliance was met only by 35 women that are included in the study, characteristics of which shows Table 2. This was due to the following reasons: (a) the majority of originally preselected patients appeared to be taking medications affecting carbohydrate and lipid metabolism; thus, they met our exclusion criteria; (b) we experienced patient drop-out of the study (some patients who started the program stopped attending follow-up visits after one, two, or three months and unfortunately, these individuals had to be removed from the study group); (c) despite declaration, some patients did not undertake a prescribed diet and/or physical activity regimen.

### 3.2. Impact of the Weight Reduction Program on Carbohydrate Metabolism

**Figure 2:**
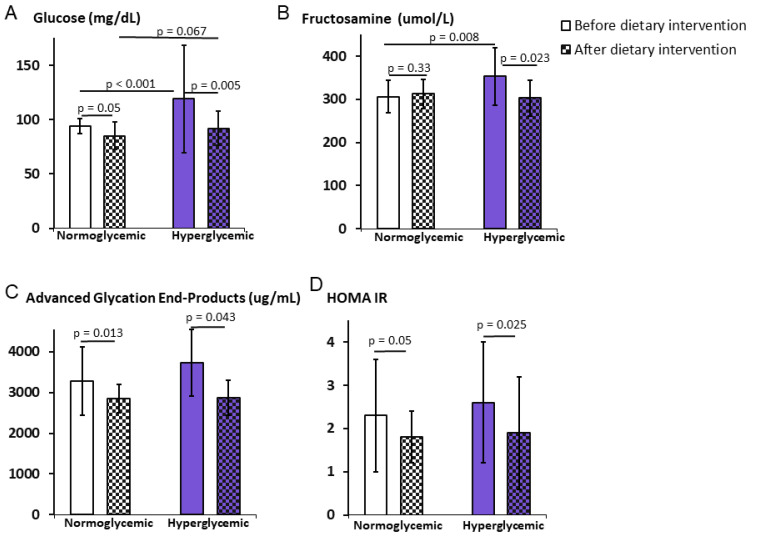
Improvement of carbohydrate profile of overweight hyperglycemic women as the result of the weight reduction program. Normoglycemic and hyperglycemic obese women were placed on a 6-month personalized weight reduction program consisting of a moderate calorie restriction and moderate exercise individualized to each patient. Glucose (**A**), fructosamine (**B**), advanced glycation products (**C**), and HOMA IR (**D**) were measured at the beginning and the end of the program. Data are mean ± SD. *T*-test significance: *p* ≤ 0.05.

Obese normoglycemic women had fasting glucose levels at 94 ± 7.0 mg/dL, while fasting glucose levels in hyperglycemic patients was moderately elevated to 119.5 ± 49.5 mg/dL (*p* < 0.001) prior to the weight reduction program. A 6-month program resulted in a significant decrease in glucose levels in both groups; however, a larger change, by 23%, was detected in hyperglycemic patients (*p* = 0.005), and levels reached normoglycemic range without difference between groups (*p* = 0.067) (Figure 2A). The initial concentrations of glycated hemoglobin (HbA1c) were within reference values at ~5.2 (%) in both groups. There were no significant changes in HbA1c concentrations after weight reduction in both study groups (Table 3). The initial levels of fructosamine of 353 ± 67.4 (µmoL/L) were higher in hyperglycemic patients compared to 306 ± 37.9 (µmoL/L) detected in normoglycemic patients (*p* = 0.008) (Figure 2B). There were no changes observed after weight reduction in the normoglycemic group; however, fructosamine levels significantly decreased to 303.0 ± 42.1 (µmoL/L) in hyperglycemic patients (*p* = 0.023) (Figure 2B).

The concentrations of AGE were elevated before the weight reduction program, reaching 3278 ± 833 (µg/mL) in the normoglycemic patients and 3725 ± 815 (µg/mL) in the hyperglycemic patients. These levels decreased significantly as the result of the program in both groups, namely to 2850 ± 345 in the normoglycemic group (*p* = 0.013) and to 2870 ± 424 in the hyperglycemic group (*p* = 0.0043) (Figure 2C). There were no statistically significant differences between these groups.

The initial concentrations of insulin were 10.2 ± 5.3 (µIU/mL) in the normoglycemic patients and 9.0 ± 3.4 in the hyperglycemic patients. There were no significant changes observed as a result of weight reduction in both groups and between the groups (Table 3). HOMA-IR index exceeded the normal range in both groups before the weight reduction program and it was 2.3 ± 1.3 in the normoglycemic group and 2.6 ± 1.4 in the hyperglycemic group. Following the program, HOMA-IR decreased in both groups, reaching the level of significance in both groups (*p* = 0.05 in normoglycemic group and *p* = 0.025 in hyperglycemic group) (Figure 2D).

### 3.3. Differential Impact of the Weight Reduction Program on Weight Loss in Normoglycemic and Hyperglycemic Obese Women

**Figure 3:**
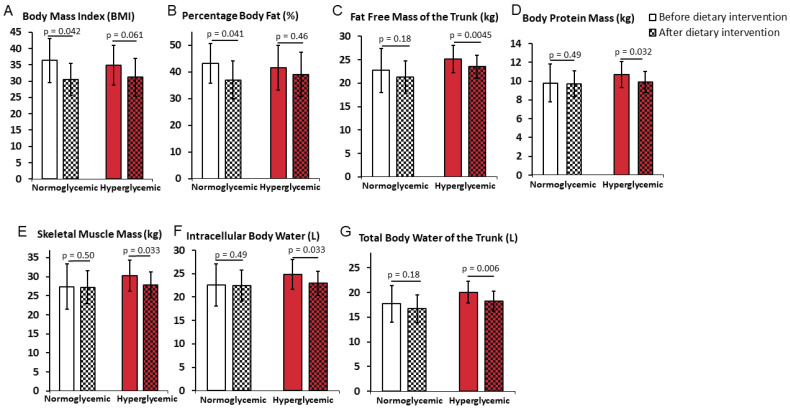
Body mass parameters that differentially changed in normoglycemic or hyperglycemic obese women placed on a 6-month personalized weight reduction consisting of a moderate calorie restriction and moderate exercise individualized to each patient. Body mass index (**A**), percent body fat (**B**), free fat mass of the trunk (**C**), protein (**D**), skeletal muscle mass (**E**), intracellular body water (**F**), and the total body water of the trunk (**G**) were measured at the beginning and the end of the program. Data are mean ± SD. *T*-test significance: *p* ≤ 0.05.

The weight reduction program resulted in a decrease in weight for both normoglycemic and hyperglycemic patients by 13% (*p* = 0.069) and 10% (*p* = 0.057), respectively (Table 4). Concurrent BMI changes in normoglycemic patients (a decrease by ~16%; *p* = 0.042) were statistically significant but not in hyperglycemic patients (a decrease by ~10%; *p* = 0.061) (Figure 3A).

Significant decreases in waist circumference (WC) by ~12% (*p* = 0.016), arm circumference (AC) by ~15%, arm muscle circumference (AMC) by ~9%, visceral fat area (VFA) by ~29% (Table 4), and percent body fat (PBF) by ~14% (Figure 3B) were observed in the normoglycemic patients as a result of the weight reduction program. On the other hand, there were no changes observed in free fat mass (FFM), skeletal muscle mass (SMM), or total body protein, as well as the parameters connected with water content in the body, such as intracellular body water (ICW) and total body water (TBW) in these patients (Figure 3C–G, respectively).

Similar to the normoglycemic patients, the weight reduction program significantly decreased WC (*p* = 0.027), AC (*p* = 0.003), AMC (*p* = 0.003) (all by 10–11%), and VFA by ~20% (*p* = 0.03) in the hyperglycemic patients (Table 4). In contrast to the normoglycemic group, there also was a significant decrease in FFM of the trunk (FFM TR) by ~7% (*p* = 0.0045) (Figure 3C), and undesired diminished values of the protein by ~7.5% (*p* = 0.032) (Figure 3D), and SMM by ~8% (*p* = 0.033) (Figure 3E), without changes in PBF (Figure 3B) in the hyperglycemic patients as a result of the weight reduction program. Significant decreases in ICW by ~8% (*p* = 0.033) (Figure 3F) and TBW of the trunk (TBW TR) by ~9% (*p* = 0.006) (Figure 3G) were also observed in the hyperglycemic patients. Overall, these results suggest that weight change in hyperglycemic patients was driven in a large part by the losses in SMM, intracellular body water, and the total body water of the trunk. There were no statistically significant changes found in extracellular body water (ECW) and bone mineral content (BMC) in both groups and between the groups (Table 4).

### 3.4. Correlations between Carbohydrate Metabolism and Markers of Body Mass

When analyzing correlations between plasma levels of carbohydrate metabolism with markers of body mass in normoglycemic patients, only limited associations have been found (Table 5). Specifically, AGE levels were positively correlated with changes in AC and AMC. On the other hand, no correlations were observed between glucose, HbA1c, fructosamine, insulin, and HOMA-IR with AC, AMC, VFA, and PBF. In contrast, strong positive correlations between AGE and VFA, between AGE and ICW, and between WC and ICW were observed in hyperglycemic patients, suggesting that changes in ICW could influence AGE levels and weight (Table 6).

## 4. Discussion

The results of the present study indicate that relatively modest lifestyle changes, such as moderate energy restriction and physical exercise, can effectively influence weight reduction and improve glucose tolerance in hyperglycemic obese women. The primary therapies for weight reduction among individuals with insulin resistance and T2D are diet and physical exercise, as they promote both weight loss and improve glucose tolerance. Indeed, it is generally accepted that energy restriction can significantly improve fasting glucose concentrations and glucose tolerance, even with relatively small changes in body weight. Such dietary intervention is generally well tolerated by obese individuals when implemented for a short period of time [32,33]. For example, it was demonstrated that blood pressure and metabolic parameters, such as lipid metabolism and glucose tolerance, can all be improved by a moderate 5–10% weight reduction [34]. Exercise, in addition to facilitating weight loss, enhances biological action of insulin, and exerts a preventive input on the incidence of T2D [35].

Abdominal obesity caused by visceral fat accumulation strongly correlates with insulin resistance and a high risk for T2D [36,37]. Therefore, the approaches to improve the glucose metabolic state are based on burning fat tissue, especially VFA. The personalized strategy implemented in the present study differs from the majority of the weight loss methodologies as it favors a long-term intervention in order to achieve and maintain sustainable changes in weight reduction and developing healthy eating and exercise habits. Moreover, longer duration of the weight reduction program, such as 6 months implemented in the present study, does not require severe dietary restrictions, which are usually difficult or even impossible to maintain for patients for a prolonged period of time.

The present study was performed on women volunteers interested in losing weight and changing lifestyles through a balanced diet and physical exercise. All patients were obese (BMI ˃ 30) with visceral fat (VFA ˃ 100) exceeding the reference values. The questionnaire revealed that patients ate unhealthily and irregularly and led sedentary lifestyles before starting the weight loss program. Part of the studied pool of patients exhibited moderately elevated glucose levels, which did not affect HbA1c levels that remained within normal range. The implemented weight reduction program consisted of a personalized dietary and exercise program and was effective as all patients lost weight and decreased several other parameters of body mass. Moreover, there were significant improvements in glucose, AGE, and HOMA-IR levels, leading to normalization of glucose metabolism. These outcomes are in line with the literature data. For example, it was demonstrated that weight loss strategies using dietary, physical activity, or behavioral interventions produced significant improvements in weight control among people with prediabetes, and a significant decrease in incidence of diabetes [38].

The observed impact of the personalized weight reduction program was strikingly different when comparing the normoglycemic and hyperglycemic patients. The most important data of the present study indicate that the weight reduction program decreased BMI only in the normoglycemic group but not in hyperglycemic women. This effect was connected with favorable loss of adipose tissue and reflected by a decrease of VFA and PBF, without any significant changes in the parameters connected to body water content (e.g., ICW) and muscle mass (e.g., body protein content and SMM). We propose that sensitivity of peripheral tissues to insulin, and thus the greater susceptibility of adipose tissue to insulin-dependent lipolysis, can be responsible for these changes. On the contrary, weight loss in patients with hyperglycemia was connected not only to the loss of VFA but also to non-fat mass loss, such as undesired decreases in SMM, free fat mass (FFM), body protein mass, ICW, and the total body water of the trunk (TBW TR). These outcomes could result from the baseline insulin resistance in these patients. Clearly, altered glucose metabolism as manifested by hyperglycemia at the start of the weight reduction program had detrimental impact on the entire weight loss process. These observations are consistent with the notion that the impact of lifestyle intervention on diabetes prevention cannot be solely ascribed to the body weight reduction [39]. It should also by noted that WC, AC, and AMC diminished in both normoglycemic and hyperglycemic patients following the weight reduction program.

While the loss of ICW in hyperglycemic patients as a result of the weight reduction program might be puzzling, the patients were encouraged to replace their diets with healthy alternatives. Thus, changing the diet and assuming a reduction in the consumption of animal saturated fatty acids (e.g., butter, cream, sausages, red meat) in favor of diet containing higher levels of healthy unsaturated fatty acids (such as fish, fresh olive oil, nuts, or seeds) can lead to remodeling of cell membranes and improvement of transmembrane transport. The effect of this remodeling may be the loss of ICW with no changes in the amount of extracellular water (ECW). In addition, the observed loss of muscle tissue (e.g., diminished SMM) could also indirectly contribute to the loss of ICW. The process of losing this tissue may, in turn, be the result of an intensification of the gluconeogenesis induced by a calorie-restricted diet. It also appears that water loss observed in patients with hyperglycemia could influence, at least in part, the observed improvements of the carbohydrate metabolic state because ICW levels exhibited a significant correlation both with changes in VFA and with AGE.

The weight reduction program resulted not only in a marked improvement of glucose levels in hyperglycemic obese women but also in a decrease in the levels of AGE in both studied groups, reflecting the overall highly beneficial impact of the strategy implemented in the present study. Lowering the concentration of AGEs is a favorable phenomenon because of high biological activity of these compounds that results in tissue damage leading to the development of diabetes and diabetic complications.

The limitations of the presented study are the following:

1. Small study groups for reasons described in the Methods section.
2. The study included only women.
3. The obtained results cannot be applied to the whole population of hyperglycemia patients, as the study conducted only patients without treatment affecting glucose and lipid metabolism.
4. The confirmation of the results in a long-term study is required.

## 5. Conclusions

The present study indicates that weight reduction based on a balanced caloric restriction diet combined with moderate exercise is more efficient in normoglycemic than in hyperglycemic obese women. Despite improvements in glucose and AGE levels, reduction in weight in obese women with hyperglycemia involved undesired loss of non-fat tissues, such as skeletal muscle mass and body protein content, as well as intracellular and total body water. These results indicate the importance of the measurements of the carbohydrate profile in overweight and obese women and introducing an early weight reduction program before the development of hyperglycemia.

## Figures and Tables

**Table 1:** General diet composition employed in the present study and assembled according to healthy nutrition recommendations of the Polish National Food and Nutrition Institute and the World Health Organization (WHO).

Nutrient	% of Total Energy Intake	Note
Carbohydrates	45–55%	Limiting intake of free sugars to less than 10% of total energy intake.
Fats	25–35%	Limiting intake of saturated fatty acids to less than 10% of total energy intake, and intake of 3–6% mono- and polyunsaturated fatty acids in the form of vegetable oils and fish oils; omega-3: omega6 1:4 (5); trans-fatty acids ˂2% of total energy.
Proteins	15–25%	Animal: vegetable sources in 1:2 ratio.
Fiber	About 1%	
Vegetable	500 g/day in portions	Low and medium glycemic index (IG).
Fruits	200/300 g/day	Low and medium glycemic index (IG).
Clean water	≥1.5–2 L/day	
Salt	˂4 g/day	

**Table 2:** Participants initial characteristic data.

Parameter	Normoglycemic Group (NG) (*n* = 15)		Hyperglycemic Group (HG) (*n* = 20)		NG vs. HG *p*	
	Before Program	After Program	Before Program	After Program	Before Program	After Program
Age [year]	51 ± 13.3		47.5 ± 7.8		0.81	
Weight [kg]	95.5 ± 21.0	83 ± 14.9	89.7 ± 16.3	80.8 ± 14.7	0.65	0,98
*p*	0.069		0.057			
BMI	36.3 ± 6.7	30.5 ± 4.9	34.8 ± 6.1	31.2 ± 5.8	0.98	0.59
*p*	0.042 *		0.061			
WC (cm)	107 ± 15	94 ± 11	103 ± 13	93 ± 14	0.89	0.51
*p*	0.016 *		0.027 *			
Glucose (mg/dL)	94 ± 7.0	85 ± 12.8	119 ± 49.5	92.0 ± 15.5	<0.001 **	0.067
*p*	0.05 *		0.005 **			

Data are expressed as mean ± SD for comparison differences between measurements before and after the weight reduction program within and between the groups. *T*-test significance: * *p* ≤ 0.05; ** *p* ≤ 0.01.

**Table 3:** Impact of the weight reduction program on glycated hemoglobin (HbA1c) and insulin levels in obese normoglycemic and hyperglycemic women.

	Normoglycemic Group (NG) (*n* = 15)		Hyperglycemic Group (HG) (*n* = 20)		NG vs. HG *p*	
Parameter	Before Program	After Program	Before Program	After Program	Before Program	After Program
HbA1 (%)	5.2 ± 0.8	5.1 ± 0.7	5.2 ± 0.5	5.2 ± 0.6	0.52	0.46
*p*	0.55		0.43			
Insulin (µIU/mL)	10.2 ± 5.3	8.6 ± 3.7	9.0 ± 3.4	8.8 ± 4.9	0.56	0.94
*p*	0.34		0.66			

Data are expressed as mean ± SD for comparison differences between measurements before and after the weight reduction program within and between the groups. *T*-test significance: *p* ≤ 0.05. Parameters of carbohydrate metabolism that changed significantly within and/or between the groups as the result of the implemented weight reduction program are shown in Figure 2A–D.

**Table 4:** Impact of the weight reduction program on selected body composition parameters in obese normoglycemic and hyperglycemic women.

	Normoglycemic Group (NG) (*n* = 15)		Hyperglycemic Group (HG) (*n* = 20)		NG vs. HG *p*	
Parameter	Before Program	After Program	Before Program	After Program	Before Program	After Program
Weight (kg)	95.5 ± 21.0	83 ± 14.9	89.7 ± 16.3	80.8 ± 14.7	0.65	0.98
*p*	0.069		0.057			
WC (cm)	107 ± 15	94 ± 11	103 ± 13	93 ± 14	0.89	0.51
*p*	0.016 *		0.027 *			
AC (cm)	38.7 ± 5.8	33 ± 3.8	39.2 ± 6.3	34.8 ± 3.2	0.38	0.16
*p*	0.012 *		0.003 **			
AMC (cm)	29.8 ± 4.5	27.2 ± 2.6	32.2 ± 5.0	29.0 ± 2.5	0.15	0.07
*p*	0.023 *		0.003 **			
VFA (cm^2^)	153.5 ± 35.3	109.2 ± 31.9	134.7 ± 32.3	110.4 ± 28.9	0.28	0.74
*p*	0.020 *		0.030 *			
FFM (kg)	49.6 ± 9.9	49.5 ± 7.4	54.2 ± 6.7	50.6 ± 6.2	0.55	0.97
*p*	0.49		0.064			
BMC (kg)	2.8 ± 0.6	2.8 ± 0.5	3.0 ± 0.4	2.9 ± 0.5	0.93	0.62
*p*	0.87		0.35			
TBW (L)	36.5 ± 7.2	36.4 ± 5.4	39.8 ± 5	37.2 ± 4.6	0.57	0.99
*p*	0.47		0.068			
ECW (L)	14.0 ± 2.7	13.8 ± 2.2	14.5 ± 2.0	14.2 ± 2.0	0.91	0.97
*p*	0.44		0.21			
ECW/TBW	0.384 ± 0.008	0.382 ± 0.011	0.38 ± 0.018	0.38 ± 0.010	0.047 *	0.76
*p*	0.58		0.20			
ECW/TBW TR	0.384 ± 0.009	0.383 ± 0.010	0.38 ± 0.017	0.38 ± 0.011	0.052	0.83
*p*	0.78		0.15			

Data are expressed as mean ± SD for comparison differences between measurements before and after the weight reduction program within and between the groups. *T*-test significance: * *p* ≤ 0.05; ** *p* ≤ 0.01. Body parameters that significantly changed in normoglycemic or hyperglycemic obese women as the result of the implemented weight reduction program are shown in Figure 3.

**Table 5:** Correlation analysis among studied parameters in obese normoglycemic women (*n* = 15) before and after the weight reduction program. The correlation is significant: * *p* ≤ 0.05; ** *p* ≤ 0.01.

Pearson’s R *p*	∆AC	∆AMC	∆VFA	∆PBF
∆Weight	0.97	0.87	0.88	0.74
	0.000 **	0.000 **	0.000 **	0.004 **
∆BMI	0.96	0.86	0.90	0.73
	0.000 **	0.000 **	0.000 **	0.005 **
∆WC	0.88	0.85	0.82	0.40
	0.000 **	0.000 **	0.000 **	0.18
∆Glucose	0.22	0.25	−0.16	−0.18
	0.47	0.41	0.59	0.57
∆HbA1	0.28	0.30	0.04	0.09
	0.35	0.32	0.88	0.78
∆Fructosamine	0.03	−0.07	−0.15	0.31
	0.92	0.83	0.62	0.33
∆AGEs	0.58	0.62	0.35	0.39
	0.039 *	0.023 *	0.22	0.19
∆Insulin	0.39	0.49	0.20	−0.01
	0.21	0.11	0.52	0.98
∆HOMA IR	0.35	0.44	0.08	−0.08
	0.26	0.15	0.81	0.80

**Table 6:** Correlation analysis among studied parameters in obese hyperglycemic women (*n* = 20) before and after the weight reduction program. The correlation is significant: * *p* ≤ 0.05; ** *p* ≤ 0.01.

Pearson´s R *p*	∆AC	∆AMC	∆VFA	∆FFM	∆FFM TRUNK	∆SMM	∆PROT	∆TBW TRUNK	∆ICW
∆Weight	0.66	0.00	0.47	−0.16	−0.07	−0.10	−0.10	−0.08	−0.10
	0.004 **	0.99	0.036 *	0.54	0.80	0.69	0.69	0.75	0.71
∆BMI	0.64	−0.02	0.48	−0.17	−0.08	−0.11	−0.11	−0.10	−0.11
	0.005 **	0.93	0.031 *	0.52	0.77	0.67	0.66	0.72	0.69
∆WC	0.54	0.07	0.51	−0.19	−0.10	−0.18	−0.18	−0.10	0.82
	0.024 *	0.80	0.023 *	0.47	0.71	0.50	0.50	0.70	0.001 **
∆Glucose	0.43	0.07	0.16	0.00	0.04	0.06	0.07	0.002	0.32
	0.09	0.78	0.49	0.99	0.89	0.83	0.78	0.94	0.28
∆HbA1	−0.09	−0.00	−0.11	0.04	−0.08	0.06	0.06	−0.10	0.43
	0.74	0.99	0.63	0.89	0.77	0.83	0.81	0.72	0.15
∆Fructosamine	0.11	−0.13	0.13	−0.09	−0.28	−0.05	−0.03	−0.31	0.06
	0.68	0.63	0.59	0.74	0.28	0.86	0.92	0.23	0.86
∆AGEs	−0.42	−0.02	0.58	−0.41	−0.45	−0.39	−0.38	−0.46	0.56
	0.10	0.95	0.007 **	0.10	0.07	0.12	0.13	0.06	0.045 *
∆Insulin	−0.17	−0.42	0.10	0.02	−0.23	0.08	0.07	−0.22	0.47
	0.54	0.12	0.69	0.95	0.42	0.79	0.80	0.43	0.13
∆HOMA IR	−0.20	−0.51	0.11	0.11	−0.30	0.18	0.18	−0.34	0.19
	0.50	0.061	0.68	0.70	0.30	0.54	0.53	0.24	0.52

## Data Availability

The database of aggregated statistics prepared for analysis is stored in secure, confidential, password-protected storage in the server of the Medical University of Silesia. The data has been anonymized. Completely deidentified records could be made available to interested persons/organizations on request at the author’s address jzalejskafiolka@sum.edu.pl.

## Abbreviations

AC, arm circumference; AMC, arm muscle circumference; AGE, advanced glycation end-products; BMI, body mass index; DCD, daily caloric demand; ECW, extracellular body water; FFM, free fat mass; HbA1c, glycated hemoglobin; HOMA-IR, homeostatic model assessment for insulin resistance; ICW, intracellular body water; IG, glycemic index; PBF, percent body fat; SMM, skeletal muscle mass; TBW, total body water; T2D, Type 2 diabetes; VFA, visceral fat area; WC, waist circumference.
